# Prediction of COVID-19 Stress among Community Dwelling Older Adults: The Role of Anxiety and Resiliency

**DOI:** 10.1093/geroni/igab046.3720

**Published:** 2021-12-17

**Authors:** Geffre Jean Francois, Dawn Carr, Natalie Sachs-Ericsson

**Affiliations:** Florida State University, Tallahassee, Florida, United States

## Abstract

Worldwide, the COVID-19 pandemic has been an unparalleled source of stress. Older adults with anxiety are vulnerable to higher levels of stress during the pandemic. However not all older adults with anxiety will experience severe stress; resiliency may decrease such negative outcomes. There have been few, if any, longitudinal studies that followed older adults before and during the pandemic. Our data of community dwelling older adults (aged 60-92) is unique in that it allowed for an investigation of psychological variables that increase and decrease negative outcomes during the pandemic. Our longitudinal study examined the influence of pre-pandemic anxiety and resiliency on the severity of COVID related stress.

**Methods:**

The pre-pandemic data was obtained in September 2018, and the pandemic data was collected in June 2020. In the baseline survey we obtained measures of anxiety and resiliency. During the pandemic we measured the severity of COVID related stressors. We hypothesized that anxiety would predict higher level of COVID-stress, whereas resiliency would be associated with decreased severity of COVID-stress. Further we predicted that resiliency would attenuate the association between anxiety and COVID-stress.

**Results:**

Using OLS regression, we found that anxiety predicted higher COVID-stress, whereas resiliency predicted lower COVID-stress. However, resiliency did not moderate the association between anxiety and COVID-stress.

**Conclusion:**

Older adults are subject to many unavoidable negative life events, such as death of family members and chronic health problems. Resiliency may help buffer against such adversities. Development of intervention programs to enhance resiliency may increase psychological resources and foster healthy aging.

